# Retrospective and Prospective Investigations about “Quatrefoil” Erythrocytes in Canine Blood Smears

**DOI:** 10.1155/2014/409573

**Published:** 2014-01-06

**Authors:** Alessandra Gavazza, Marianna Ricci, Martina Brettoni, Biancaurora Gugliucci, Anna Pasquini, Daniela Rispoli, Nicola Bernabò, George Lubas

**Affiliations:** ^1^Department of Veterinary Sciences, University of Pisa, via Livornese Lato Monte, San Piero a Grado, 56124 Pisa, Italy; ^2^Department of Comparative Biomedical Sciences, University of Teramo, Piazza Aldo Moro 45, 64100 Teramo, Italy

## Abstract

The presence of unusual two RBCs patterns (so-called “quatrefoil RBCs,” qRBCs) on canine blood smears at Optical Microscope (OM) was seen during routine evaluation of CBCs. Two consecutive retrospective investigations were arranged including about 7,000 CBCs and clinical records and laboratory data from dogs showing qRBCs. Few samples with qRBCs were prepared for Scanning Electron Microscope (SEM). qRBCs were found in 6.89% (139 of 2016) and 8.47% (133 of 1569) of dogs and in 3.89% (154 of 3,958) and 4.47% (138 of 3,081) of CBCs (some dogs were tested more than once). Statistical analysis was significant for age groups (Chi squared, *P* < 0.0001), decreased total leukocyte and neutrophil counts (ANOVA, *P* < 0.0001), RBCs anisocytosis, polychromasia, and Howell-Jolly bodies (ANOVA, *P* < 0.018, <0.005, and <0.003, respectively). qRBCs were distributed in the area of feathered edge and at the smear side of body-feathered edge area in blood films. SEM ruled out the possibility of an optical illusion or an accidental overlap. qRBCs are associated with ageing of dogs, total leukocyte and neutrophil counts, and RBC anisocytosis, polychromasia, and Howell-Jolly bodies. Few hypotheses were discussed to explain the origin and meaning of this RBC arrangement.

## 1. Introduction

Evaluating blood films whenever a CBC is requested validates data generated by hematology analyzers and provides information related to RBCs, WBCs, and platelets (PLTs). Evaluation of RBCs morphology is a critical step in blood smear evaluation adding important information [1, 2]. Morphologic evaluation of RBCs from a well-made blood smear can sensitively detect abnormalities in size and hemoglobin concentration as well as provide information about abnormal cell shape (poikilocytosis) and the presence of inclusions, not detectable even with the most advanced analysers. This procedure is generally underutilized by practitioners largely because of the lack of confidence in preparing a well-made blood film and in being unable to identify important abnormalities. However, no other hematology procedure provides more valuable information yet requires so little additional time (two-three minutes) and expense. Indeed, a good experience is needed to evaluate a blood smear and in particular “abnormal” cells [3, 4].

A variety of causes can modify RBC shape including regenerative response, altered metabolism (iron and lipid above all, but also electrolyte depletion), oxidative injury, immune-mediate damage, mechanical fragmentation, sepsis, toxins, and drugs, and also artifactual agents like temperature, humidity, and errors in smear preparation (e.g., anticoagulant excess, drying artifacts), the so-called preanalytical errors [5, 6]. These causes are not mutually exclusive, there are a lot of possible intermediate forms and the same morphological features may be seen in various physiological or pathological states. The real abnormalities may be nonspecific and associated with numerous mechanisms or highly specific and essentially diagnostic of inherited or acquired disease [1, 7].

Since 2010, we documented the appearance of a particular arrangement of two erythrocytes occurring in blood smears of dogs. Since then we prepared a couple of theses in Veterinary Medicine degree and three short communications on this topic. We called this abnormality “Quatrefoil RBCs” (qRBCs) or “Pisa Cross RBCs” as they resemble the appearance of a quatrefoil or the flag of Pisa community back in the middle age times [8–12].

The aim of this study was to summarize investigations carried out about the presence and the occurrence of qRBCs on canine blood film and to relate them to specific condition or disorder or other abnormalities in laboratory results.

## 2. Materials and Methods

The qRBCs were occasionally found during blood smears evaluation at optical microscope (OM) over the years, but a specific box to report them routinely in CBC results was added only in May 2009, in order to store this information in the data base.

Two time-consecutive investigations were arranged to study the appearance and consistency of qRBCs.

In the first survey 3,958 CBCs were included collected from 2,016 dogs in the period from May 2009 to October 2010 (18 months). For each dog sex (in both sexes neutered dogs were included), age, and breed were reported. The classes of ages used were puppies from 4 to 12 months, adults from 13 months to 6 years old, and elderly over 7 years old. Blood samples were collected in K3-EDTA tubes. CBC parameters were assessed using a veterinary impedance cell counter (HeCoVet, SEAC, Calenzano, Florence, Italy) and included RBC count, hemoglobin (Hgb), hematocrit (Hct), mean corpuscular volume (MCV), mean corpuscolar hemoglobin (MCH), mean corpuscolar hemoglobin concentration (MCHC), PLT count, mean platelet volume (MPV), plateletcrit (PCT), red cell distribution width (RDW), platelet distribution width (PDW), and WBC count. Blood films were prepared with the slide (wedge) method as quickly as possible (generally within one hour from the blood collection) and rapidly air-dried to minimize morphologic changes. The smears were stained with Diff-Quik (Dade Behring, SpA, Milano, Italy) according to procedures indicated by the manufacturer. Blood films were used to perform the WBC differential count, the morphological evaluation of RBCs, WBCs, and PLTs, and the PLT estimation as well. Cellularity, cell identification, staining, and cell distribution were evaluated both at 20x and 100x magnification in the area between the body and the feathered edge of the blood film. This area is usually known as where about 50% of red cells should be touching one another. The CBC blood film scanning was performed by all authors, except Nicola Bernabo'.

154 CBCs showing qRBCs and their respective dogs from which the blood samples were collected were further investigated taking in account their clinical records including all the laboratory data carried out at the same time (serum biochemical profile including total proteins, albumin, cholesterol, glucose, total calcium, phosphates, total bilirubin, urea, creatinine, iron, alanine amino transferase, aspartate amino transferase, alkaline phosphatase, gamma glutamyl transferase, and serum protein electrophoresis fractions) and their health or disease status inferred. Dogs without the presence of qRBCs served as control group.

During this study a specific investigation was arranged to view the qRBCs with higher magnification, using the Scanning Electron Microscopy (SEM) (JEOL/JSM-5410 SEM, JEOL Ltd., Tokyo, Japan). Initially, according to Goel et al., 2006, liquid samples were prepared from the blood of five dogs who presented qRBCs more than once with 3–5 drops of blood immediately added to 5 mL of glutaraldehyde (2.5% in phosphate buffered saline or PBS. After 3 hours at 4°C, the samples were centrifuged (2,700 rpm for 10 minutes) to obtain RBC pellets. The RBC pellet was washed twice in PBS and then smeared on glass slide of 1.5 × 1.5 cm previously treated with polylysine. Then smears were dehydrated with progressive alcohol solutions (30-50-70-90-95%) and finally twice in absolute alcohol. The preparations were further dehydrated with a critical point-dried and covered by a thin layer of gold. Then, the following procedure from blood smear of three dogs who presented qRBCs more than once was established: RBC quatrefoils were identified on blood smears previously fixed with methanol only (using the Diff-Quik fixative solution) and examined at OM at 20x magnification. Then, the areas of interest were isolated from the blood smears and the glass slides were cut out to fit SEM required size (1.5 × 1.5 cm) and directly covered by a thin layer of gold (avoiding the dehydration procedure as above described) to be processed with the SEM [13].

In the second survey, 3,081 CBCs were included collected from 1,569 dogs in the period December 2010-2011 (13 months). For each dog the variables sex, age, and breed were collected as the previous study. Blood samples were collected in K2-EDTA tubes. CBC parameters were assessed using a veterinary laser cell counter (Pro-Cyte, Idexx Laboratories, Italy) and included RBC count, Hgb, Hct, MCV, MCH, MCHC, PLT count, MPV, PCT, RDW, PDW, WBC count, automated leukocyte differential count, and reticulocyte count. Blood films were prepared as described before; only the staining method was different as an automatic slide stainer was used (Wescor 7150 Aerospray, Delcon, Milano, Italy) with the May-Grundwald Giemsa stain.

From all blood smears examined belonging to this second survey, other erythrocyte morphological abnormalities detected during the routine evaluation (grading and recognition according to Weiss 1984 [14]) were collected, analyzed, and compared with the CBCs without the presence of qRBCs serving as control group (1984) [14]. In addition, a sample of 64 CBCs with qRBCs over 138 totally, using different dogs, was examined to localize and count the qRBCs in the smear (the scanning rules were described before).

Occasionally, qRBCs were also seen in CBCs from other species. During the first survey 676 cats, 259 horses, and 8 wild boars were examined, while in the second survey 514 cats, 20 calves, and two bears were evaluated.

In order to appreciate the spatial configuration of qRBCs after SEM exam, a three-dimensional (3D) draw draw was arranged.

### 2.1. Statistical Analysis

The statistical analysis has been carried out with the following tests: Chi-squared for sex and age, Fisher's exact test for breed, and one-way ANOVA (Mann-Whitney) for CBC parameters and all RBC abnormalities recorded and for results from serum biochemical profile and serum protein electrophoresis.

## 3. Results

In Table 1 the results of the initial survey including 2,016 dogs and 3,958 CBCs are reported. The qRBCs were found in 139 dogs (6.89%) and in 154 CBCs (3.89%). Sex was not a statistically significant variable, while the comparison for age groups was significant (Chi squared, *P* < 0.0001). The breeds most represented were mixed, Labrador, German Shepherd, and Golden Retriever (without any statistical significance). It should be noted that qRBCs were also found and recorded at the same time in ten cats (10/676), two horses (2/259), and one wild boar (1/8).

The difference between the number of CBCs performed and number of dogs included in the study is due to the repetition of more than one CBC in the same dogs. The same difference is occurring in the CBCs showing qRBCs. Indeed, samples with quatrefoil RBCs were 154 in 139 dogs because 14 out of 139 dogs (10.1%) presented this RBC pattern more than once (usually twice; one dog showed qRBCs in 3 sequential CBCs). qRBCs were not observed in every single blood film of these subjects, but 5 out of 14 dogs showed qRBCs in consecutive smears.

In Table 2 the results of the second survey including 1,569 dogs and 3,081 CBCs are reported. The qRBCs were found in 133 dogs (8.47%) and in 138 CBCs (4.47%). Sex was not a statistically significant variable, while the comparison for age groups was significant (Chi squared, *P* < 0.0001). The breeds most represented were mixed, German Shepherd, Golden Retriever, and Labrador (without any statistical significance). It should be noted that qRBCs were also found and recorded at the same time in two cats (2/514), one calf (1/20), and one bear (1/2), while none qRBCs were seen in horses.

In Table 3, based on clinical records and laboratory data for dogs presenting qRBCs in the first survey the assignment by their health status as non-diseased or affected by an organ system illness or disorder was possible. It should be noted that 9/139 dogs were diseased in more than one system or affected by more than one disorder. Unfortunately, we were not able to assign 33/139 dogs either to healthy or diseased group. qRBCs were detected mostly in dogs affected by neoplasia (mainly malignant lymphoma and mastocytoma), followed by otherwise healthy animals and in dogs affected by various gastrointestinal disorders (mainly enteropathies of different origin). The ANOVA test performed on values obtained from CBC, biochemical profile, and serum protein electrophoresis parameters revealed that total leukocytes count (qRBC group 3.08–26.38, median 8.6 × 10^9^/L; control group 4.10–31.9, median 10.2 × 10^9^/L), absolute neutrophils count (qRBC group 2.19–23.48, median 6.58 × 10^9^/L; control group 2.94–26.8, median 6.96 × 10^9^/L), and neutrophils percentage (qRBC group 42.6–88.1, median 71%; control group 45–94, median 76%) were statistically (*P* < 0.0001) lower in dogs with qRBCs in comparison to the control group.

In Table 4 findings of other standard erythrocyte morphological features in 3,081 CBCs without qRBCs (control group) and in 138 CBCs with qRBCs (qRBC group) are reported. The ANOVA statistical evaluation pointed out a significant result for anisocytosis and polychromasia and the appearance of Howell-Jolly bodies between the two groups. It should be also pointed out that the erythrocyte morphological features were observed as occasionally single or generally in combination with each other.

**Figure 1:**
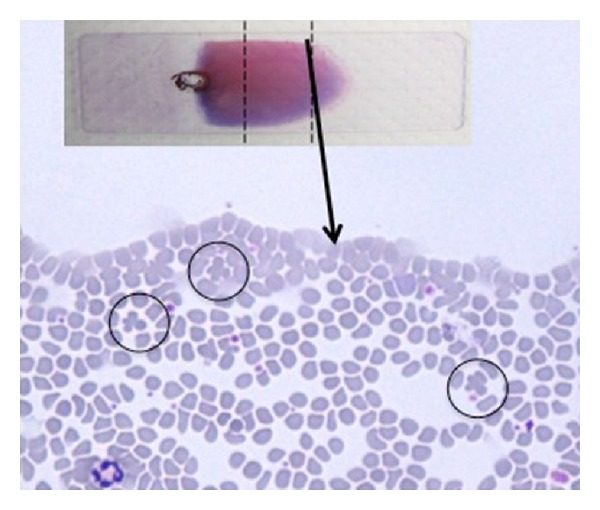
Few qRBCs seen (black circle) at the smear side adjacent to the body-feathered area (20x, May-Grundwald Giemsa).

**Figure 2:**
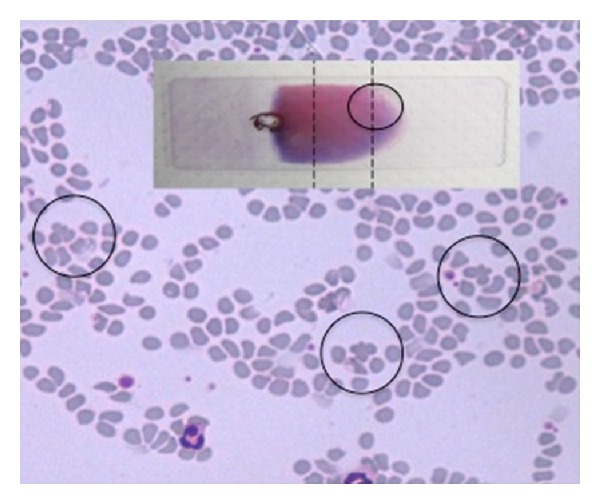
Few qRBCs seen (black circle) in the feathered edge area of the blood film (20x, May-Grundwald Giemsa).

**Figure 3:**
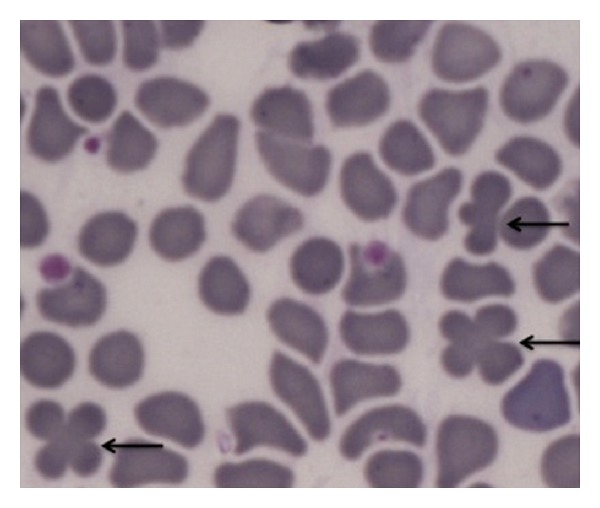
In this blood film at HPF (100x, May-Grundwald Giemsa) three qRBCs (black arrows) can be easily seen.

In Table 5 the investigations about the position of qRBCs in 64 blood films further scrutinized for this purpose are reported as follows:

- in most of the cases only one qRBC was observed (41/64) in all smear scrutinized;
- qRBCs were equally seen at the feathered edge area and at smear sides adjacent to the body-feathered area (see Figures 1 and 2);
- in few cases qRBCs were seen in both previously cited areas;
- the maximum number of qRBCs seen in the same HPF (100x) was 3 (see Figure 3).

**Figure 4:**
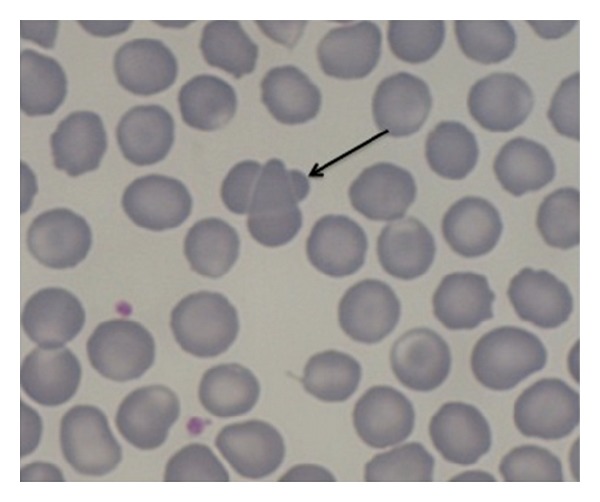
In this blood film at HPF (100x, May-Grundwald Giemsa) an intermediate form of qRBCs is easily seen (black arrow).

Finally, in 23/64 blood films “intermediate forms” of qRBCs have been observed. The intermediate forms of qRBCs are recognized as two RBCs partially overlapping and with their shapes still rounded (not sharpened, as resembling acanthocytes). Blood films showing one qRBCs and intermediate forms of qRBCs were 7/23 (30.4%), while those with more than one qRBCs along with intermediate forms were 16/23 (see Figure 4).

**Figure 5:**
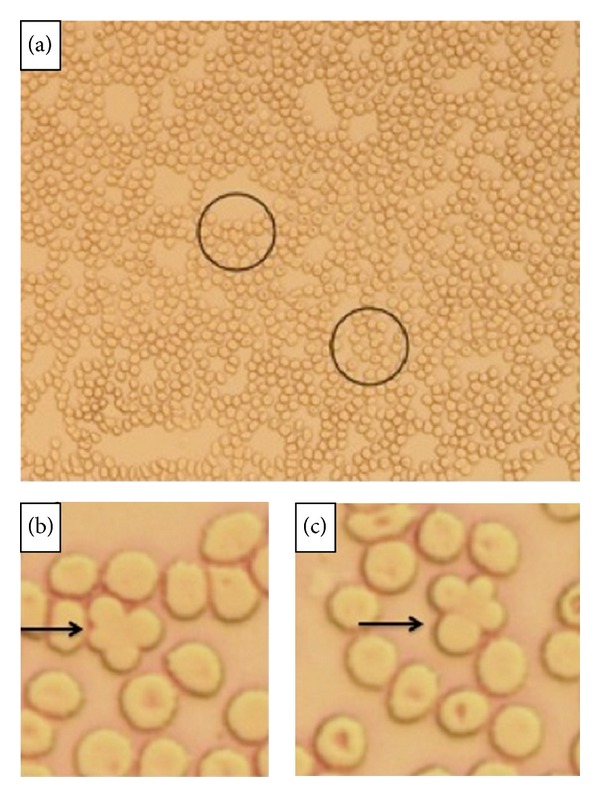
Unstained blood film in the feathered edge area where it can be seen two qRBCs at low (20x, black circle) (a) and high magnification (100x, black arrow) (b) and (c). These samples were used for SEM investigation.

**Figure 6:**
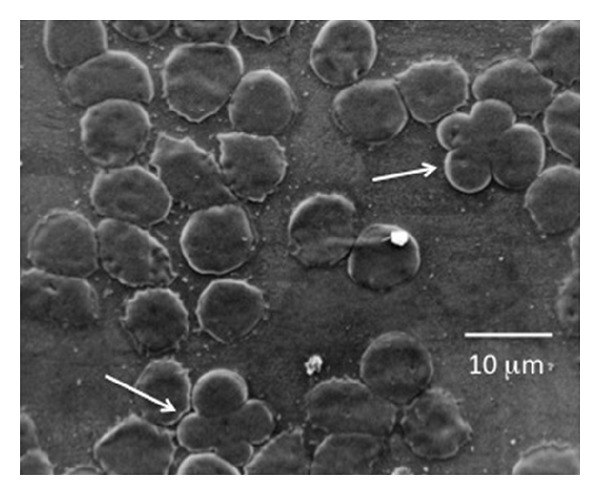
Two qRBCs (white arrow) in blood film at SEM (150x, 10 kV).

**Figure 7:**
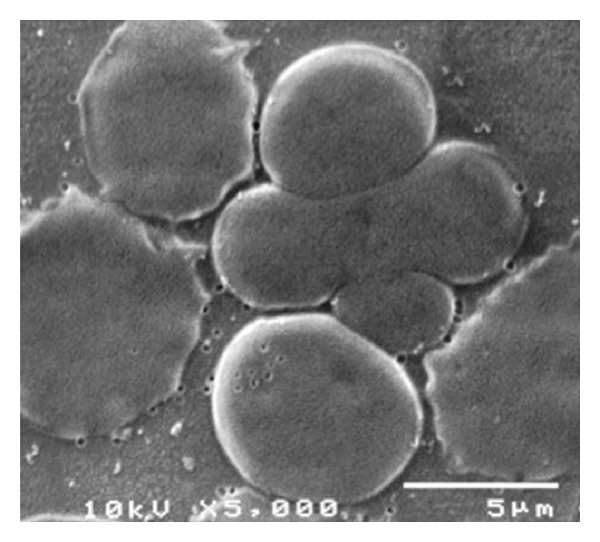
The magnification of one qRBC in blood film at SEM (×5000, 10 kV).

**Figure 8:**
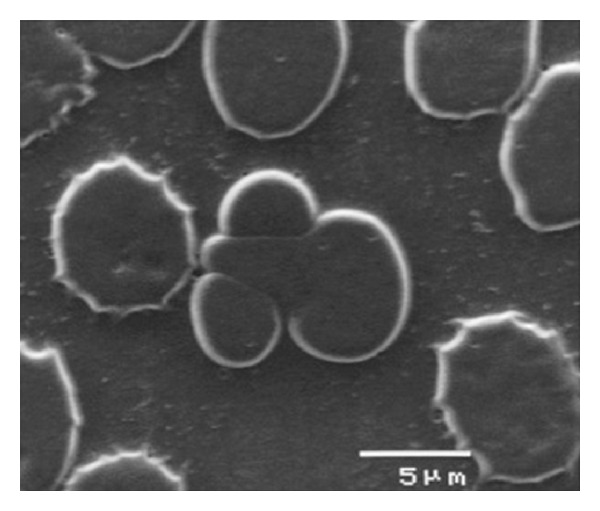
The magnification of one “incomplete” qRBC in blood film at SEM (×5000, 10 kV).

**Figure 9:**
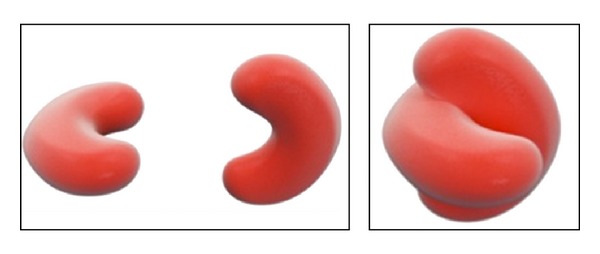
Supposed spatial arrangement of qRBCs in a three-dimensional (3D) draw.

Two different preparations to observe qRBCs at SEM were arranged, the former from glutaraldehyde-treated blood samples and the latter from blood already smeared on glass slide. Both techniques were successful to prepare intact RBCs to view at SEM. Unfortunately, blood smears examined at SEM prepared from glutaraldehyde-treated blood samples were unsuccessful to observe any qRBCs. Therefore, the examination at SEM of blood smear where qRBCs were already seen and localized was the best choice to observe qRBCs at high magnification. This closer observation ruled out the possibility of an optical illusion or an accidental overlap (see Figures 5, 6, 7, and 8). Looking at the inner edges the impression that two erythrocytes were “embraced” was confirmed, and a spatial arrangement was drawn in 3D (see Figure 9).

**Figure 10:**
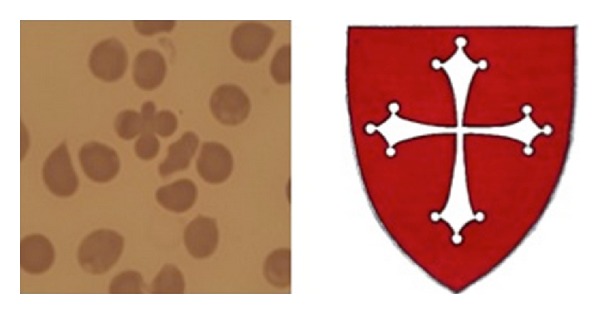
qRBCs are resembling the Pisa middle ages cross.

The further name assigned to qRBCs was Pisa cross RBCs as this modification is highly resembling the Pisa cross of middle ages (see Figure 10).

## 4. Discussion

During regular scanning of blood films the occurrence of quatrefoil RBCs was largely incidental, single, and only in few smear areas. When a careful examination was performed in these slides multiple findings of quatrefoil RBCs were quite common. Our results of qRBCs findings are probably underestimated, because usually blood smear evaluation takes few minutes and all the information needed to complete the morphological evaluation included in the CBCs are collected only from few side areas.

The two consecutive investigations arranged showed a slight difference in prevalence values considering the number of dogs (139/2,016, 6.9% vs. 133/1,569, 8.5%) or number of CBCs (154/3,958, 3.9% vs. 138/3,081, 4.5%). This difference could be related to the awareness, attention, training, and experience of the several cytopathologists performing the blood films evaluations, which were increasing day by day in their routine work.

In both surveys the comparison of the qRBCs appearance and the variables sex, age, and breed was significant only for the age classes arranged, with this modification more observed in elderly dogs. It should be noticed that among the breeds more represented in both surveys were mixed dogs and then Labrador, German Shepherd, and Golden retriever versus German Shepherd, Golden retriever, and Labrador. Furthermore, the sample processing was slightly different in the two surveys; besides, the vial anticoagulant for collecting blood was K3-EDTA versus K2-EDTA and the staining method for blood films was Diff-Quik versus May-Grundwald Giemsa, but these conditions seem unremarkable for the appearance of qRBCs. Indeed, qRBCs were recognized in both surveys even if with different prevalences. As noted, qRBCs were also observed in blood samples and their respective blood films collected without any anticoagulant.

The search to find a specific disorder or disease related to the appearance of qRBCs was unsuccessful; however, it should be noted that dogs affected with cancer (mainly lymphoma and mastocytoma) and otherwise healthy were more represented in subgroups arranged in Table 3. The association of qRBCs in dogs with lower leukocyte count (absolute) and neutrophil count (absolute and percentage) in comparison to control population of dogs without qRBCs is interesting and should be confirmed in further extensive studies.

The relationship with other variations in the RBCs such as volume, color, and shape which are routinely scanned during a blood film evaluation was statistically significantly associated in anisocytosis and polychromasia and the appearance of Howell-Jolly bodies with the appearance of qRBCs. All these abnormalities are generally occurring when there is an erythroid stimulation.

The qRBCs arrangement was equally found in the feathered area and in the smear side area adjacent to the body and feathered area, in the area of optimal thickness for light microscopic examination, and where artifacts and distortion forces to cells are minimized. In addition, the preferred areas for evaluation are those where erythrocyte central pallor is observed and cells are round [15]. From results obtained qRBCs are generally isolated findings and there are few samples with more than 2-3 qRBCs and they are mainly located at the smear sides. The sites where the majority of qRBCs was found are compatible with the spreading of larger cells at the side of the smear.

The preparation of blood for SEM was fruitful only using smears in which qRBCs were already localized at OM. This technique was so far a new procedure never described before in dogs and gave very nice results. Indeed, we were able to document qRBCs spatial arrangement ruling out both an optical illusion or accidental overlap of two RBCs due to smear (shear) forces during the preparation of slides. The glutaraldehyde-treated blood samples could be negative for qRBCs searching mainly because the rarity of qRBCs in the blood and because the aliquot of blood smeared was without any qRBCs or otherwise the glutaraldehyde-treatment could detach the two RBCs involved in the qRBCs arrangement.

The qRBCs represent an adhesion between two erythrocytes, which is different from rouleaux (organized linear arrays or chains of RBCs) and mere agglutination (unorganized clumping or clusters). Rouleaux and agglutination could share common causes, that is, the increased concentration of globulin proteins, and species differences should also be taken in account as noted by Windberger et al., 2003, which pointed out different viscosity in entire blood and plasma and RBC aggregation [16].

The quatrefoil shape observed with OM seems to be formed by two erythrocytes laid in a sort of “embrace.” The size of the arrangement compared to a single RBC supported the hypothesis as well as the impression derived from the observation of inner edges (as shown in Figures 3, 4, 6, and 7).

Several hypotheses were advanced to explain this particular RBC arrangement.

The very first hypothesis was that qRBCs represented an overlap artefact. In a few of the initial observation of blood smears with qRBCs, artifactual dacryocytes (same direction) on the feathered edge were observed, which overlapped other RBCs (as shown partially in Figure 4). This hypothesis was soon rejected because it was not confirmed with more experience in seeing slides with qRBCs. Indeed, it was clear that the dacryocyte arrangement presented pointed ends and were morphologically different from real qRBCs having rounded shape.

Another artifact hypotheses due to EDTA was considered. Although EDTA does not distort blood cells, making it ideal for hematology application, it has some effects on cell membrane, which could influence RBC adhesivity. The use of EDTA as an anticoagulant increased the osmotic fragility of red blood that reflects the functionality of the erythrocytes' membranes [17]. Studying human ghost membrane with SEM, it was proved that, after exposure to EDTA, the entire membrane surface was covered with long, thin extrusions called stromalitic forms (about 460 per cell) only visible with SEM. These extrusions seem to be related to the characteristic of chelator (calcium binding), because exposure to ionized calcium abolished the EDTA-induced stromalitic form. Calcium plays a role in some cellular activities, influences adhesivity, and confers rigidity and structural cohesiveness to membrane components. The existence of stromalitic forms indicate that the membrane surface is unstable or weakened or that its surface tension, that affect adhesion between RBCs, has been altered by the chelators [18]. Therefore, quatrefoil RBC could be artifactual arrangement due to EDTA chelating activity that may lead to a dysfunctional adhesivity, together with forces applied during the blood smear preparation. The concentration of EDTA in relation to the blood collected and the type of EDTA in the tube could also play a role. In our study we did not note any difference using K2 or K3 EDTA anticoagulated samples.

The alternative hypotheses to artifactual issues considered the physical point of view.

Generally, RBCs show discocyte appearance (biconcave) and they have a greater surface area than is required to enclose their volume (normal equilibrium); they can, consequently, bend and fold without increasing surface area. Lacking a 3D cytoskeleton, RBCs maintain their shape and mechanical integrity through a spectrin-dominated, triangular 2D network attached to the cytosolic side of their plasma membrane. This semiflexible filament network, along with the surface tension of the bilayer, contributes to the elastic moduli of the composite membrane. Under particular shear flow conditions (nonequilibrium), RBCs can tumble and roll and can assume different shapes, such as elongation into an ellipsoid while undergoing tank-treading (TT) motion [19, 20].

From the biophysical point of view the RBC consists of an outer elastic cell membrane and inner viscous fluid and its motion under viscous shear flow is affected by membrane elastic property. Indeed, the RBC exhibits two motions. The TT motion is a rotation of the membrane around the interior, maintaining a constant shape, and this occurs when fluid external forces are greater than membrane elastic resistance force because the absence of cytoskeletal elements in the cytoplasm allows the membrane and cytoskeleton to differentially revolve around the cytoplasm [20, 21]. Alternatively, the RBC motion turns (transition) to tumbling motion, an overall rotation of the entire cell like a rigid body. This transition is an oscillation of the entire RBC together with the membrane TT motion and occurs when the maximum elastic resistance force is comparable to the external fluid share force [7, 20].

Few papers in experimental models show the existence of different threshold shear in which RBC changes its motion (TT or tumbling) in relation to the viscosity of the medium where are suspended. The active role played by the RBC cytoskeleton and its periodic deformation is reflected in potential energy oscillations that are the result of each point on the RBC membrane being unique, a property also known as “shape memory.” The cytoskeleton is intrinsically attached to the membrane and these oscillations are due to the alternate contraction and stress relaxation of the cytoskeleton. However, TT motion is observed in a flow regime in which theoretical models would predict only tumbling. This apparent violation of theoretical prediction may be attributed to the fact that the altered morphology causes both the work done and the dissipation to be different and indicates that TT motion is pervasive even in folded and deformed erythrocytes, conditions that occur when such erythrocyte flow through narrow capillaries [20, 22, 23].

Finally, cell adhesion is a fascinating process that plays a key role in many situations of biological and medical interest, but whereas erythrocytes are fairly smooth at the submicrometer level, nucleated cells are studded with a variety of protrusions, blebs, ruffles, microvilli, or lamellipodia with complex mechanical behavior and an obvious influence on adhesive interactions. The blood circulation in vessels is accompanied with cell-cell and cell-wall collisions, which could lead to adhesion. The blood cell adhesion is a short-range force originating from molecular forces such as electrostatic, hydration, steric, Van der Waals, and specific chemical bonding. The surface tension affects adhesion between RBCs and it is related to the number of adhesion molecules, most of which are transmembrane proteins attached to the cytoskeleton [24].

RBCs aggregation starts with small aggregates (typically doublets) that can be broken by Brownian collisions, which cause the adhering cells to move apart, but new aggregates also form in a dynamic equilibrium. Stronger adhesion of RBCs should displace this equilibrium to higher concentration of aggregates. The extent of aggregation is determined by opposing forces: the aggregation induced by the presence of macromolecules and the disaggregation induced by the negative surface charge and the flow-induced shear stress. RBCs in the presence of plasma proteins or other macromolecules may form aggregates, normally in rouleaux formations, which are dispersed with increasing blood flow [25, 26].

In our results, rouleaux were higher in the control population (14.5%) than in dogs with qRBCs (9.0%) (see Table 4). Therefore, the hypothetical adhesion in qRBCs probably has to be found into mechanisms different from both rouleaux and agglutination formation and so different from the mere presence of macromolecules. In the blood flow, cell-cell contact times are rather short when RBC's “bump” into each other, but if contact is made slow enough, repulsive molecules are expected to depart from the contact region or gather into restricted areas, as predicted in view of theoretical considerations and suggested by electron microscopic evidence leading to an adhesion [24, 27].

It should be kept in consideration that the shear rate in microcirculation is low and guarantees blood-cell and cell-blood exchanges, promoting slow approach between RBCs end an hypothetical local adhesion. From the “shape memory” it is known that each point of RBC membrane is unique and it can still be supposed that circulating RBCs adhering by a small area (for contact forces or specific adhesion molecules) together under the influence of tank-treading motions and shear flow forces may lead to a “slip” of one cell membrane to the other, distortion of cells with biconcavity loss, and forming an unusual circulating qRBCs. Multiple findings in the same dogs (14 dogs, 10.1%) may indicate an individual predisposition to qRBCs formation [23, 24, 27].

The negative result to find qRBCs in the liquid SEM samples do not invalidate this theory because glutaraldehyde affects RBC membrane composition of proteins even in small concentration (0.0005%) with formation of protein aggregates that parallel when the concentration increases. Changes in the spectrin and other proteins of cytoskeleton network may lead to changes of the erythrocyte shape and size as well as unleash an adhesion between two erythrocytes [28, 29].

## 5. Conclusion

Based on the results obtained and discussed it was not possible to identify with certainty the origin and the meaning of the “quatrefoil” modification, despite the fact that its effective occurrence in blood smears was confirmed. Few interesting statistical data were documented such as correlation with the ageing, total leukocyte and neutrophil counts, and few other RBC features (anisocytosis, polychromasia, and Howell-Jolly bodies). In several informal interviews with other veterinarian cytopathologists and/or hematologists we had the confirmation that qRBCs in canine blood films were occasionally observed as a curiosity. The same interview with human cytopathologist and/or hematologist did not give the same results as they never saw such RBCs conformation. In addition, it would be interesting to follow and monitor dogs that show qRBCs in their blood especially those with a high concentration of qRBCs in order to avoid the missing recognition of this RBC pattern as it occurred in dogs where is only occasionally found.

## Figures and Tables

**Table 1:** Sex, age, and breed of dogs included in the initial survey (2,016 dogs—139 dogs with qRBCs = 6.89%) 3,958 CBCs were investigated and 154 CBCs showed qRBCs (3.89%).

	No. of dogs	% Dogs
Sex		
Males	73	52.5
Females	66	47.5
Chi squared	*P* > 0.05	
Age		
Puppies (0–12 m)	22	15.8
Adults (13 m–6 y)	39	28.0
Elderly (>7 y)	78	56.2
Chi squared	*P* < 0.0001	
Breed		
Mixed	40	28.8
Labrador	18	12.9
German Shepherd	11	7.9
Golden Retriever	8	5.8
Poodle	5	3.6
Rottweiler	5	3.6
Setter	5	3.6
Boxer	4	2.9
Other breed	43	30.9
Fisher exact test	*P* > 0.05	

m: month; y: year.

**Table 2:** Sex, age, and breed of dogs included in the second survey (1,569 dogs—133 dogs with qRBCs = 8.47%) 3,081 CBCs were investigated and 138 CBCs showed qRBCs (4.47%).

	No. of dogs	% Dogs
Sex		
Males	70	52.6
Females	63	47.4
Chi squared	*P* > 0.05	
Age		
Puppies (0–12 m)	14	10.5
Adults (13 m–6 y)	45	33.8
Elderly (>7 y)	74	55.7
Chi squared	*P* < 0.0001	
Breed		
Mixed	41	30.8
German Shepherd	16	12.0
Golden Retriever	9	6.8
Labrador	8	6.0
Setter	7	5.3
Border Collie	5	3.8
Boxer	4	3.0
Epagneul Breton	4	3.0
Other breed	36	27.2
Fisher exact test	*P* > 0.05	

m: month; y: year.

**Table 3:** Assignment of 139 dogs with qRBCs in healthy or diseased subgroup.

	No. of cases	% Cases
Healthy	25	16.9
Unknown	31	20.9
Neoplastic*	30	20.2
Gastrointestinal*	17	11.5
Dermatological	10	6.7
Endocrinal	7	4.7
Neurological	7	4.7
Renal*	6	4.2
Hepatic	4	2.8
Reproductive	3	2.0
Cardiac	3	2.0
Respiratory	3	2.2
Bone and Muscle	2	1.4

	148	

*Dogs included in this group were affected by another disease in organ system or disorder.

**Table 4:** Distribution of standard erythrocyte modification findings (according to [12]) in two groups of dogs without quatrefoil RBCs or with quatrefoil RBCs.

	Group without qRBCs (%) No.: 3,081	Group with qRBCs (%) No.: 138	*P*
Anisocytosis	61.3	70.7	0.018
Polychromasia	25.1	35.1	0.005
Poikilocytosis	34.5	37.8	Ns
Echinocytes	20.1	22.7	Ns
Rouleaux	14.5	9.0	Ns
Howell-Jolly Bodies	11.6	19.4	0.003
Dacryocytes	6.4	9.0	Ns
Elliptocytes (ovalocytes)	6.1	7.8	Ns
Schistocytes	4.5	3.2	Ns
Acanthocytes	3.9	3.9	Ns
Keratocytes	2.1	2.3	Ns
Eccentrocytes (hemighosts)	1.2	2.3	Ns
Spherocytes	0.7	0.6	Ns
Target cells	0.7	1.3	Ns

Ns: not significant.

**Table 5:** Quantitation and position of qRBCs in blood films from a sample of 64 CBCs.

No. of cases subtyped for qRBCs seen in the smear	qRBCs totally seen in the smear	No. of cases where qRBCs were seen in			Maximum no. of qRBCs seen at HPF (100x)
		Feathered edge area	Smear sides area (body-feathered edge adjacent)	Feathered edge and smear sides areas	
41	1	26	15	0	1
4	2	1	2	1	2 (1 case)
6	3	1	5	0	3 (4 cases)
6	4	1	4	1	3 (2 cases)
2	5	0	2	0	2 (1 case)
3	6	0	2	1	3 (2 cases)
2	7	0	1	1	3 (1 case)

64	—	29	31	4	—
